# Inspection of Trivalent Chromium Conversion Coatings Using Laser Light: The Unexpected Role of Interference on Cold-Rolled Aluminium

**DOI:** 10.3390/s20082164

**Published:** 2020-04-11

**Authors:** Joerg Rischmueller, Yannic Toschke, Mirco Imlau, Mareike Schlag, Hauke Brüning, Kai Brune

**Affiliations:** 1 Department of Physics, Osnabrueck University, Barbarastrasse 7, 49076 Osnabrueck, Germany; jrischmu@uni-osnabrueck.de (J.R.); ytoschke@uni-osnabrueck.de (Y.T.); 2 Fraunhofer Institute for Manufacturing Technology and Advanced Materials IFAM, 28359 Bremen, Germany; mareike.schlag@ifam.fraunhofer.de (M.S.); hauke.bruening@ifam.fraunhofer.de (H.B.); kai.brune@ifam.fraunhofer.de (K.B.)

**Keywords:** laser-based inspection, interference, layer model, trivalent chromium conversion coating, cold-rolled aluminium, rough surface, AA3003

## Abstract

Laser-based inspection of trivalent chromium conversion coatings on rough, cold-rolled aluminium substrates is studied from a basic physics perspective by means of angle and wavelength dependent measurements. As a result, we show that the correlation between the scattered laser light and the coating weight of the conversion layer is dominated by the phenomenon of interference. The combined experimental and numerical approach of our study is based on an appropriate layer model which was developed from a set of reference measurements of confocal microscopy, electron microscopy and X-ray photoelectron spectroscopy. The aluminium alloy AA3003 with a trivalent chromium conversion coating serves as an example. Our derived model is capable to reconstruct the reflectance of a laser beam at grazing incidence even for a pronounced surface roughness of $R_{q}\approx 300\;nm$, for different coating thicknesses less than 70 nm corresponding to coating weights between zero and 0.5 g/m${}^{2}$ and for laser wavelengths from 405 nm to 785 nm. In our discussion we outline the possibility to transfer the results to other aluminium alloys and/or other metallic substrates, as well as their potential for industrial applications such as 100% inline-capability, costs, velocity and ruggedness.

## 1. Introduction

It is impossible to imagine today’s everyday life without functional coatings modifying and enhancing the surface properties of different materials. Specially tailored for specific applications they serve a broad range of functionalities, for example the improvement of historic urban districts [1], drag reduction by means of riblets in the aircraft industry [2,3], increased biological compatibility in medical technologies [4,5] and corrosion protection in the light metal industry. One specific type of the latter used in this study are so called conversion coatings which can be applied as part of a pre-treatment process, thus providing excellent protection against environmental degradation and improving the adhesion of subsequently applied coatings at the same time. One noteworthy and, in the last decade, widely used form of passivation is the highly corrosion resistant chromate conversion coating (CCC) consisting of a backbone of trivalent Cr(III) and hexavalent Cr(VI) chromium [6,7]. Because of the toxic and carcinogenic attributes of the latter, Europe’s REACH agreements 2017 (Regulation on Registration, Evaluation, Authorisation and Restriction of Chemicals) restricted the use of conversion coatings containing Cr(VI) in general. While those restrictions have led to further investigations of suitable alternatives, one of the most promising substitutes based on Cr(III) and Zr(IV) (SurTec^®^ 650-chromitAL TCP., SurTec International GmbH, Bensheim, Germany) is used exemplarily in this study. According to the literature, several papers have already been published using SurTec^®^ 650 as passivation agent on different aluminium alloys backing our choice [8,9,10,11,12,13]. Additionally, researchers like Kim et al. applied this agent on the commonly used AA3003 (here on aluminium foil) which serves as our substrate of choice [14]. While most publications focus on an improved understanding of the chemical formation process, the optimization of treatment conditions in terms of increased corrosion protection or use in situ spectroscopic ellipsometry (SE) as a tool for studying the growth kinetics of TCP coatings [15,16], this study primarily focuses on coating thickness measurements. Note that it is a challenging task for SE as well as for reflectometry to investigate rough samples in general. Exemplarily, Lehmann et al. addressed this problem by presenting a new optical roughness model for thin films with a peak-to-valley distances between 10 nm and 60 nm, which still is significantly smaller than our cold-rolled and coated industrial specimens ranging in the micron regime [17]. At this point it is noteworthy that Siah et al. showed that it is possible to extract film thicknesses and refractive indices only at discrete wavelengths due to depolarization effects when investigating thin films deposited on rough Si-wafer [18].

**Figure 1:**
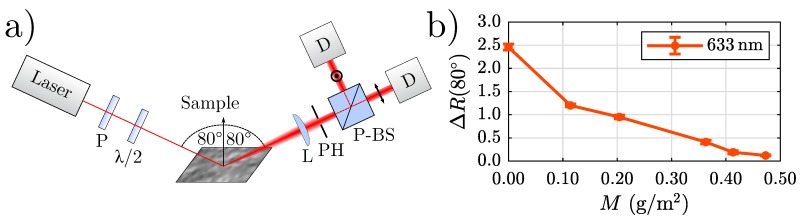
(**a**) Sensor concept for investigating the trivalent chromium conversion (TCC) coatings using $\theta_{\mathrm{in}}=\theta_{\mathrm{out}}=80{}^{\circ }$ and a polarization direction of $45{}^{\circ }$ (P: polarizer; λ/2: waveplate; L: lens, f=5cm; PH: pinhole; P-BS: polarizing beam splitter; D: Si-photodiode). (**b**) Corresponding results [19].

An optical method capable of detecting and distinguishing between different thicknesses of trivalent chromium conversion (TCC) coatings on rough cold-rolled AA3003 aluminium substrates has already been presented in the literature by us showing that our suggested measurand $\Delta R\left(80{}^{\circ }\right)=R_{s}\left(80{}^{\circ }\right)/R_{p}\left(80{}^{\circ }\right)$ correlates strongly with the estimated coating weight *M* [19]. This finding is briefly reviewed in Figure 1. The left panel of Figure 1 schematically depicts the sensor concept: The light beam of a helium-neon laser (λ=632.8nm, $P_{0}=1mW$) is guided grazingly (angle of incidence amounts to $\theta_{\mathrm{in}}=80{}^{\circ }$ with respect to the surface normal) onto the sample. The polarizer (P) only transmits linearly polarized light and the λ/2 waveplate rotates the polarization direction to $45{}^{\circ }$. The specularly reflected light is focused with a lens (L, f=5cm) and guided through a pinhole (PH). Using a polarizing beam splitter (P-BS), the light beam is separated into two perpendicularly polarized parts and detected by two Si-photodiodes (D, OSD50). It is important that the detection takes place in specular direction, meaning $\theta_{\mathrm{out}}=\theta_{\mathrm{in}}=80{}^{\circ }$. Figure 1b shows the result of a measurement performed with the setup depicted in Figure 1a. Six cold-rolled AA3003 samples with different coating weights *M* have been investigated. Our measurand $\Delta R(80{}^{\circ })$ decreases strongly with increasing coating weight.

In this publication we address the underlying physical origin of the previously found correlation.

## 2. Materials and Methods

### 2.1. Materials

The samples investigated in our study are briefly presented in this chapter. As mentioned above, the popular general-purpose aluminium alloy AA3003 (see for example [20] for more information) serves as substrate without using a polishing procedure after the rolling process. Thus, the surface exhibits pronounced roughness and possesses scratches as well as bends. A summary of the parameters used for the TCC coating process is shown in Table 1. Note that the uncoated specimen Ref goes through all steps despite the passivation step 10 (marked with bold printing).

Table 2 shows the variation in time and temperature for the passiviation step to fabricate 5 samples with different coating weights ranging from 0.114 g/m${}^{2}$ to 0.473 g/m${}^{2}$ determined by gravimetric analysis. Accordingly, the coated specimens are entitled as PT1 (Pretreatment) to PT5.

### 2.2. Methods

For the purpose of understanding the physical origin of the connection between our measurand $\Delta R(80{}^{\circ })$ and the applied coating weight *M*, we first address possible light-matter interactions taking place at the cold-rolled rough samples. In general, several physical mechanisms like scattering on top of the surface, absorption occurring inside the coating, scattering at embedded defects and at the substrate–coating interface, as well as interference effects, that all together interfere and affect the measured signal. Due to these considerations we used three reference measurements for characterizing the samples: First, laser scanning confocal microscopy (LSCM, Keyence GmbH, Neu-Isenburg, Germany, model *VK9700*) for determining the topography, especially the surface roughness described by $R_{q}$. Second, scanning electron microscope (SEM, Thermo Fisher Scientific Inc., Oregon, USA, model *FEI Helios NanoLab 600*) and a focused ion beam section to gather information about the coating thickness and uniformity. Third, X-ray photoelectron spectroscopy (XPS, Kratos Analytical Ltd, Manchester, U.K., model *Axis Ultra*) to evaluate the chemical composition of the TCC coatings at the air–coating interface.

**Figure 2:**
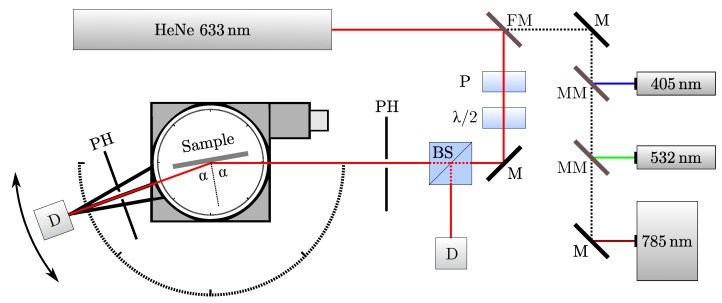
Experimental setup for investigating the samples with adjustable wavelength, angle of incidence, angle of detection and polarization state (P: polarizer; λ/2: waveplate; PH: pinholes; BS: beam splitter; D: Si-photodiode; FM: flip-mirror; MM: magnetic mirrors; M: mirrors).

The experimental setup for the optical measurements is schematically sketched in Figure 2. For investigating the angular dependency of our measurand $\Delta R(\theta_{\mathrm{in}})$, we used two independent rotating motors (Newport Corporation, Irvine, CA, USA, model *M-URM80CC*) as goniometer. Thus, we can precisely control the angle of incidence $\theta_{\mathrm{in}}$ and detect the specularly scattered light with $\theta_{\mathrm{out}}=\theta_{\mathrm{in}}$. By means of magnetic mirrors (MM) and a flip mirror (FM), it is possible to couple four different lasers into the setup and thus investigate the wavelength dependency of ΔR(λ). We used 405 nm (Picotronic GmbH, Koblenz, Germany, model *DD405*), 532 nm (Picotronic GmbH, Koblenz, Germany, model *DD532*), 633 nm (helium-neon laser, $P_{0}=1mW$) and a temperature stabilized laser diode (Thorlabs Inc., Newton, USA, model *L785P090*) emitting 785 nm. In the main beam path, the light from one of the lasers is guided through a combination of polarizer (P) and λ/2 waveplate to set the polarization state to s- or p-polarization. A beam splitter (BS) reflects a certain amount of light to a reference diode (D) and thus enables us to compensate laser fluctuations. The light reflected from the sample is detected by a second photodiode after passing through two pinholes (PH), one in front and one behind the investigated specimen. It should be mentioned that the sensor concept depicted in Figure 1a operates with optimal parameters regarding wavelength and angle of incidence for investigating the AA3003 samples, as will be shown in the progress of this manuscript.

## 3. Experimental Results

In the first part of this chapter we present the results of the reference measurements, enabling us to perform an appropriate characterization of the samples. Second, the findings of the optical setup presented in Figure 2 are shown, in particular the wavelength and angular dependence of ΔR.

### 3.1. Reference Measurements

#### 3.1.1. Roughness: LSCM

**Figure 3:**
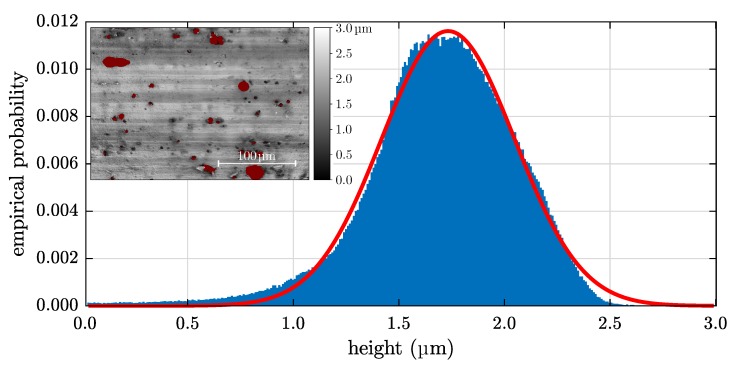
Height distribution of the Ref sample calculated from the laser scanning confocal microscopy (LSCM) image in the inset.

Figure 3 shows the height distribution of the reference sample generated from the LSCM image presented in the inset. The examined area has a size of 212 × 282 μm${}^{2}$ while the red masked pickling pits covering 3.5% of the total area are excluded from the calculation of $R_{q}$. Grooves running parallel to the long side of the specimen are clearly visible and are a result of the rolling process. Note that the height distribution is sufficient to a Gaussian distribution as shown by the fitted red line except of a slight skewness. This result holds in general for cold-rolled specimens [21].

The roughness and peak-to-valley distance of all investigated samples are presented in Table 3. The determined $R_{q}$ values range between 260 nm and 290 nm indicating that the passivation step has only slight influence on the roughness.

#### 3.1.2. Coating Weight and Thickness: FIB Section

**Figure 4:**
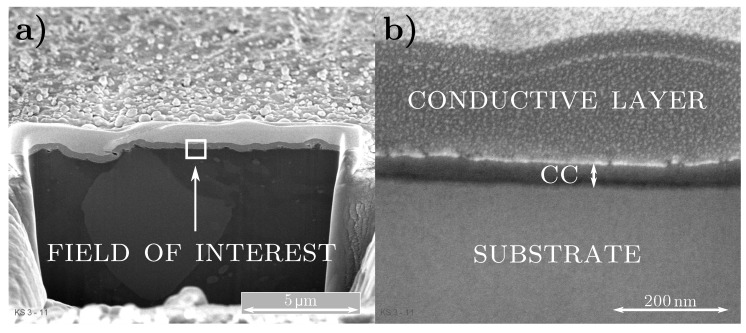
Scanning electron microscope (SEM) images of specimen PT5, (**a**) macroscopic view and (**b**) zooming in the area marked by the white rectangle.

For gathering information about the coating thickness and its uniformity, we used SEM measurements after preparing a small section with a focused ion beam. The left part of Figure 4 shows a macroscopic view on the prepared section of specimen PT5 with highest coating weight. A white rectangle indicates the part that is investigated in Figure 4b with higher resolution. The conversion coating follows quite precisely the underlying substrates height variations and exhibits only small deviations in its thickness. The latter one is estimated to be smaller than 70 nm. Note that we provide SEM measurements of the other samples within Supplementary Material.

#### 3.1.3. Chemical Composition of the TCC Coatings: XPS

To evaluate the chemical composition of the TCC coatings at the air–coating interface we used XPS measurements. We examined the samples at two different positions and present the results after averaging in Table 4. As expected, neither Zr nor Cr is detectable on the reference sample, while approximately 7 at% Zr and 3.5 at% Cr are determined for all samples with a conversion coating. Due to the sampling depth of XPS of approximately 10 nm only the topmost surface is analysed, therefore quite similar results are obtained for the samples PT1 to PT5. Also, due to this effect the Al signal strongly decreases and the Cu signal vanishes completely when a coating is applied. In addition, the carbon content of the Ref sample with 24 at% is in a normal range (known as ubiquitous carbon), as the samples were not sputtered. However, a significant increase of the carbon content after passivation can be observed. We suggest that the reason for this is due to carbon-containing compounds within SurTec^®^ 650 since other authors have also observed an accumulation of carbon within the conversion layer [8,9]. The high oxygen content is due to oxide layer formation, whereby primarily chromium(III)–zirconium(IV)–aluminium mixed oxides are formed within the conversion layers [13]. The different proportions of fluorine, nitrogen and sulfur result from a slightly inhomogeneous layer formation, so that even at the two different measured spots of the same sample differing values are present to a certain extent.

**Figure 5:**
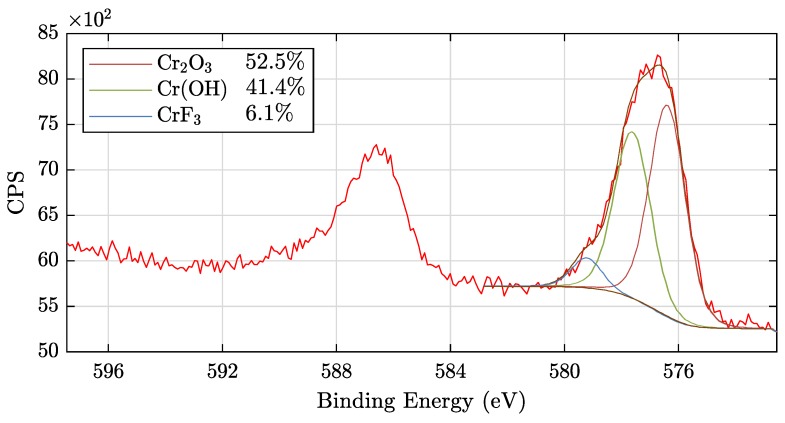
High resolution XPS spectrum and curve fitting for the Cr 2p photoelectron region for specimen PT4.

Figure 5 shows a high resolution XPS spectrum and the result of the fitting procedure of the Cr2p3/2 peak for the sample PT4 as an example. The chromium species include Cr${}_{2}$O${}_{3}$ ( 576.4 eV), Cr(OH) ( 577.6 eV) and CrF${}_{3}$ ( 579.2 eV) accounting for 52.5%, 41.4%, and 6.1% of the total chromium, respectively. Chromium fluoride has been used for the fitting procedure because of the high amount of fluorine inside the conversion layer as suggested in reference [9]. We note that we can not completely exclude the (residual) presence of Cr(VI) species. In any case, the details of the chemical content do not enter the analysis of the optical signals, as will be shown below. Furthermore, we provide high resolution XPS spectra of the other samples within Supplementary Material.

### 3.2. Optical Measurements

**Figure 6:**
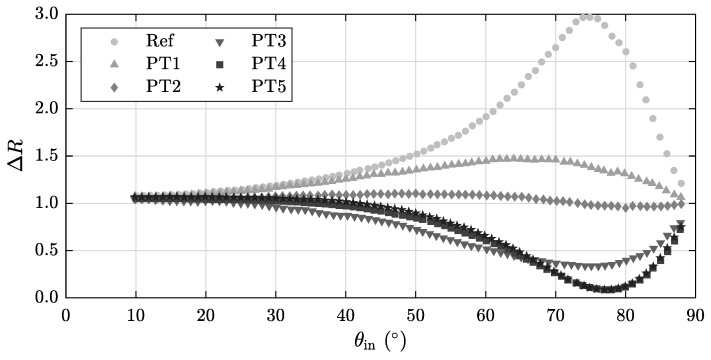
Angle-dependency of $\Delta R(\theta_{\mathrm{in}})$ using a wavelength of 633 nm.

This section deals with the dependency of our measurand $\Delta R(\theta_{\mathrm{in}},\lambda )$ on the angle of incidence $\theta_{\mathrm{in}}$ and the wavelength λ using the setup described in Figure 2. The results obtained for 633 nm are shown in Figure 6. While all samples head for the same value of $\Delta R(\theta_{\mathrm{in}})$ at the periphery ($0{}^{\circ }$ and $90{}^{\circ }$), they differ most strongly between $60{}^{\circ }$ and $80{}^{\circ }$ which results in all specimens being clearly distinguishable. It is noticeable that the thicker coatings PT4 and PT5 strive towards a pronounced minimum at approximately $78{}^{\circ }$, while the other samples reach interim values corresponding to their increasing layer thickness.

**Figure 7:**
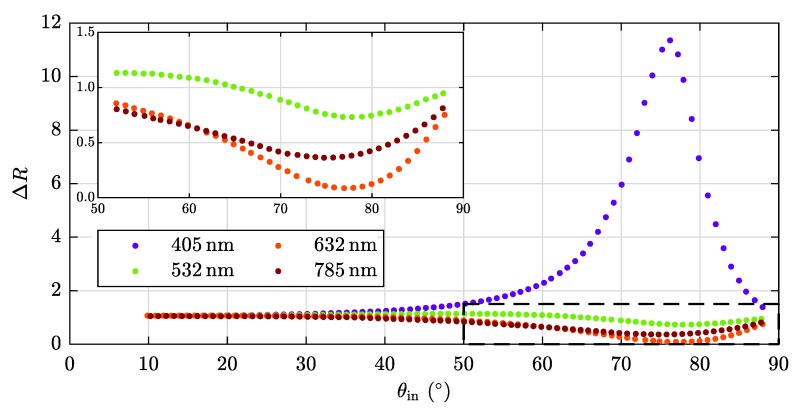
Wavelength-dependency of our measurand ΔR(λ) using the exemplary sample PT5.

For having a closer look at the influence of the used wavelength λ on ΔR, we couple four different laser systems into our optical setup. Exemplarily, the results of our specimen with maximal coating weight, PT5, are presented in Figure 7. Strikingly, the blue curve describing ΔR(405nm) shows a pronounced maximum at $\theta_{\mathrm{in}}\;\approx 76{}^{\circ }$, whereas the other curves head for a minimum. The black rectangle depicts the area enlarged in the inset. It is noteworthy that ΔR(633nm) strives to the lowest value of all curves.

## 4. Discussion

The inspection of TCC coatings on cold-rolled aluminium substrates by means of our previous described setup provides a strong relationship between our measurand ΔR and the applied coating weight *M*. Furthermore, the necessity of a carefully chosen wavelength and angle of incidence is shown. In the following section we answer the question regarding the physical origin of the previous described correlation.

### 4.1. Three Layer Model for TCC Coatings on Cold-Rolled Aluminium

First, we focus on the mathematical description of the light scattering with the uncoated specimen Ref. The LSCM measurements from Section 3.1.1 proved Gaussian distributed height values and thus the applicability of a model described by Beckmann and Spizzichino [22]. Here we confine ourselves to the most important statements, however, for more details we refer to the Appendix B.

Beckmann and Spizzichino derived that the averaged, squared electric field ${<|E|}^{2}>$ consists of two summands, one for the diffusely scattered light and one for the specularly scattered light:

$$\begin{array}{c} {<|E|}^{2}>=\mathrm{Spec}+\mathrm{Diff} \end{array}$$

Restricting ourselves to grazing incidence ($\theta_{\mathrm{in}}\mapsto 90{}^{\circ }$) as well as detecting with $\theta_{\mathrm{in}}=\theta_{\mathrm{out}}$, the specular part gets dominant and thus Spec≫Diff holds leading to ${<|E|}^{2}>\approx \mathrm{Spec}$. To consider the polarization of the incoming light one can write

$$\begin{array}{c} <|E_{k}{|}^{2}>={\mathrm{Spec}}_{k}\approx \mathrm{Spec}\;\cdot \;R_{k}, \end{array}$$

while *k* denotes s-polarized or p-polarized light and $R_{k}$ the corresponding reflectance described by Fresnel. Dividing the reflectance of both polarization states it follows:

(1) $$\begin{array}{c} \Rightarrow \frac{<|E_{s}{|}^{2}>}{<|E_{p}{|}^{2}>}=\frac{\mathrm{Spec}\;\cdot \;R_{s}}{\mathrm{Spec}\;\cdot \;R_{p}}=\frac{R_{s}}{R_{p}}=\Delta R \end{array}$$

Equation (1) defines in general our measurand ΔR as $R_{s}/R_{p}$ and is used as fitting function for the Ref sample in Figure 9. Furthermore, note that an important assumption for the validity of the Beckmann and Spizzichino model is the neglect of shadowing affects. Considering the peak-to-valley distance of $D_{h}=1.60\mu m$ and a groove distance of 3.3 μm (see Appendix A) this assumption is reasonable for our samples.

**Figure 8:**
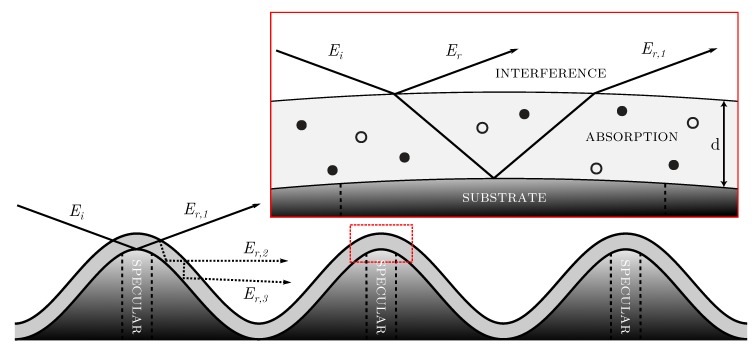
Sketch of the interaction of an incident electric field $E_{i}$ with the investigated substrate. The inset illustrates the use of a grazing incidence: Incident light strikes primarily the mountains of the height distribution where the curvature of the latter can be neglected for $E_{r}$ as well as for $E_{r,1}$, thus leading to an approximately flat surface.

For extending the description to coated cold-rolled aluminium, we take the results from the LSCM measurements as well the FIB section into account. The passivation step affects the roughness $R_{q}$ marginally (see Table 3) and the TCC coating follows quite precisely the underlying substrates height variations combined with only small deviations in its thickness (see Figure 4). These findings are schematically depicted in Figure 8.

In particular, Figure 8 shows that for grazing incidence the incoming light described by the electric field $E_{i}$ interacts primarily with the mountains of the height profile. In the red marked inset we focus into a peak of such a mountain and depict the physical description for the coated specimen: One part of $E_{i}$ is reflected at the air–coating interface, the other part transmits into the layer obeying Snell’s law. The latter is reflected at the coating–substrate interface, propagating again through the TCC layer. Reaching the coating–air interface, the beam again is split into two parts, one transmitting into air (denoted as $E_{r,1}$) and the other one staying in the coating due to reflection. Note that the rays $E_{r,2}$ and $E_{r,3}$, which are reflected several times within the conversion layer, experience an additional change in direction. As a result, they do not contribute to the specularly reflected part and thus are not considered in the mathematical description of the coated samples. This fact is different to the model used in reflectometry and ellipsometry where multiple reflections are considered in a way that smooth specimens are required for the validity of this description. Note that for simplicity the surface is drawn as a sinusodial function and thus does not exhibit Gaussian distributed height values.

The electric field that interacts with the detector in specular direction is described as a superposition of two waves:

(2) $$\begin{array}{cc} E_{r} & =r_{0,1}E_{i}e^{i(kz-\omega t)} \end{array}$$

(3) $$\begin{array}{cc} E_{r,1} & =t_{0,1}r_{1,2}t_{1,0}E_{i}e^{i(n_{\mathrm{TCC}}^{\prime }\frac{\omega }{c}z-\omega t)}e^{-n_{\mathrm{TCC}}^{\prime \prime }\frac{\omega }{c}z}e^{i\delta }, \end{array}$$

where $E_{i}$, $E_{r}$ and $E_{r,1}$ denote the incoming, first and second reflected wave, respectively, *k* denotes the wavenumber of the reflected waves, *z* the position, ω the angular frequency, *t* the time, $r_{n,m}$ and $t_{n,m}$ the reflection coefficient and transmission coefficient for the interface of material *n* and *m*, respectively, *c* the speed of light, $n_{\mathrm{TCC}}^{\prime }$ and $n_{\mathrm{TCC}}^{\prime \prime }$ the real part and imaginary part, respectively, while δ describes an additional phase shift. The latter results from the optical path length occurring inside of the layer. If there is an additional phase shift Δ at an interface, this must also be considered. In summary, one gets for the phase shift:

(4) $$\delta =\frac{4\pi \left(n_{\mathrm{TCC}}^{\prime }+i\;\cdot \;n_{\mathrm{TCC}}^{\prime \prime }\right)\cos\left(\theta_{in}^{\prime }\right)}{\lambda }+\Delta$$

Finally, the expression for ΔR for samples with TCC coating results in:

(5) $$\Delta R=\frac{<|{\left(E_{r}+E_{r,1}\right)}_{s}{|}^{2}>}{<|{\left(E_{r}+E_{r,1}\right)}_{p}{|}^{2}>}$$

### 4.2. Evaluation of the Proposed Model

**Figure 9:**
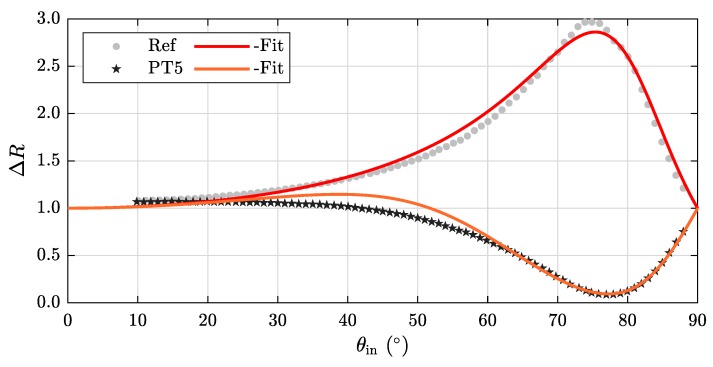
Experimental data sets for specimen Ref and PT5 together with a corresponding fit function described in Equations (1) and (5), respectively. We exclude data points ranging to $60{}^{\circ }$ for the fitting procedure due to the fact that the mentioned equations are valid only for high angles of incidence.

In Figure 9 we apply the above described model to our experimental results: Shown is the data set already presented in Figure 6 for specimens Ref and PT5 combined with a fitting curve based on the derived Equations (1) and (5), respectively. As described before, our model holds only for high angles of incidence. Therefore we exclude data points for smaller angles than $\theta_{\mathrm{in}}=60{}^{\circ }$ to ensure an accurate fitting procedure.

**Figure 10:**
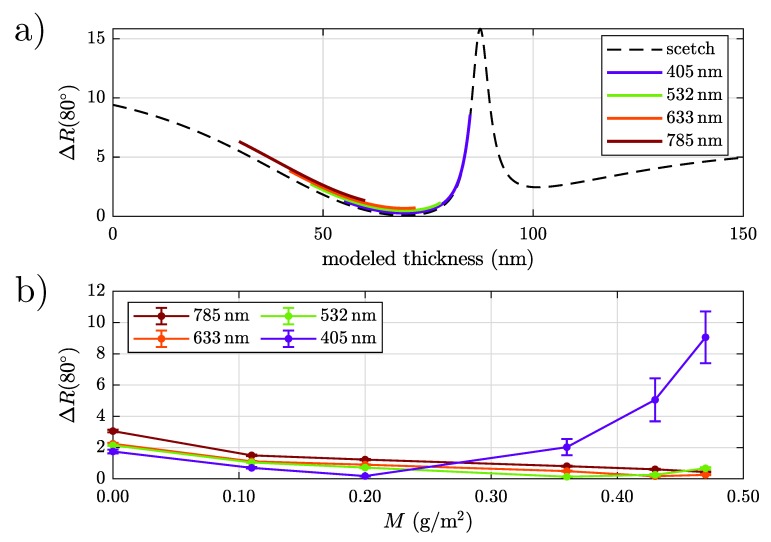
$\Delta R(80^{\circ })$ (black dotted curve) is calculated as a function of a modeled thickness in (**a**). In (**b**) the experimental results for our specimen using four different wavelengths are shown. The data fit well to the predicted behavior in (a).

We proceed the described fitting procedure for all investigated samples leading to the results presented in Table 5. Note that the substrate’s refractive index is calculated by using the reference sample leading to $n_{\mathrm{Sub}}^{\prime }=1.90\pm 0.05$ and $n_{\mathrm{Sub}}^{\prime \prime }=3.24\pm 0.06$ at a wavelength of 633 nm. This result is thereupon inserted in the fitting procedure of the coated specimen. The modeling yields a similar layer thickness of about 70 nm for the samples PT4 and PT5, although the coating weight of PT5 ( 473 g/m${}^{2}$) is greater than the one of PT4 ( 413 g/m${}^{2}$). The reason for this effect is the sinusoidal shape of ΔR (see Figure 10a): Both samples are located very close to the minimum, which leads to the fact that variations in the layer weight cause only slight changes in ΔR. If a better differentiation in the range of the higher coating weights is necessary, a larger wavelength must be used. It is noticeable for thinner coatings that the real part $n_{\mathrm{TCC}}^{\prime }$ increases while the imaginary part $n_{\mathrm{TCC}}^{\prime \prime }$ decreases at the same time. This means the thicker conversion layers absorb slightly, whereas the applied layers of the samples PT1 and PT2 can be regarded as absorption-free. In the case of the PT1 sample, it is not possible to restrict the real part to a precisely determined interval. In general, the estimated parameters of the thicker coatings are defined more precisely than those of the thin layers.

### 4.3. Determination of the Optimal Wavelength

Figure 10a depicts the theoretical behavior of our measurand $\Delta R(80^{\circ })$ in black as a function of a modeled layer thickness using a wavelength of 633 nm. The change in phase occurring due to a different wavelength used has been converted into a corresponding layer thickness equivalent for 633 nm. For example, a 50 nm layer measured at a wavelength of 405 nm is converted to a modeled thickness of 78 nm in Figure 10. First, $\Delta R(80^{\circ })$ is a decreasing function and reaches its minimum at a layer thickness of approximately 70 nm. This effect occurs due to a strong decrease of the signal in s-polarization. At 87 nm $\Delta R(80^{\circ })$ strives to a pronounced maximum because of a strong decrease of the detected p-polarized light. The colored schemes indicate the expected result for $\Delta R(80^{\circ })$ at the corresponding wavelengths for the specific coating weight of our specimen: For 785 nm and 633 nm $\Delta R(80^{\circ })$ is a monotonically decreasing function while this is not valid for 532 nm and 405 nm.

Figure 10b shows our experimental results and proves the behavior of $\Delta R(80^{\circ })$ predicted in Figure 10a. The data’s trend can be described well although the absolute values differ from the predicted ones especially for small coating weights. This fact occurs because the model is derived for coated specimens and thus is not valid for uncoated samples. Using smaller wavelengths, especially 405 nm irradiating coated specimens, first the s-polarized part $R_{s}$ strives for a minimum leading to the same result for $\Delta R(80^{\circ })$. For increasing layer thickness $R_{S}$ leaves the minimum while the p-polarized part $R_{p}$ decreases strongly leading to high values of $\Delta R(80^{\circ })$. It is important in respect of a sensor concept that $\Delta R(80^{\circ })$ provide an unambiguous relationship to the layer thickness and thus to the coating weight. Therefore, we used 633 nm while detecting with 532 nm or 405nm is not expedient due to the fact that different coating weights lead to the same value for $\Delta R(80^{\circ })$. Note that these results can be easily extended to other conversion coatings requiring only metal as substrate for inducing a phase shift between s- and p-polarization, respectively. Thus our model, used in particular for our specimen in Figure 10a, provides a calibration curve enabling the selection of an optimal angle of incident and wavelength for differing combinations of substrate and conversion coating.

In summary, the physical origin of the correlation between our measurand $\Delta R(80^{\circ })$ and the applied coating weight *M* can primarily addressed to interference between the two rays depicted in Figure 8. Due to the high roughness $R_{q}$ as well as peak-to-valley distances $D_{h}$ in the micron regime, this states a quite surprising fact.

### 4.4. Conclusions and Future Work

The understanding of the strong correlation of our measurand ΔR with the coating weight *M* creates a foundation to transfer our optical method to other aluminium alloys, as well as differing metallic substrates, and thus enables the comprehensive use in the light metal industry. For a successful implementation of the optical setup (from a user’s perspective) the following requirements have to be fulfilled: First, a metallic substrate is required to induce a phase difference between s- and p-polarized light for investigating coatings thinner than 70 nm. Second, the applied coating must be mostly intact and following the height variations of the substrate with uniform thickness for the most part. Finally, absorption within the conversion layer should be limited, so that a sufficient penetration depth is achieved and destructive interference can efficiently occur. Depending on composition of the conversion layer, this requirement can define a lower limit for the usable wavelength. With respect to the laser source it is noteworthy that optical powers below 1 mW can be used, which makes the setup fall in line with laser protection requirements. Due to the grazing incidence the illuminated area on the sample is enlarged thus reducing the minimal spatial resolution. As an upside, the subsequently imposed areal averaging in combination with the s/p-normalization achieved by using $\Delta R=R_{s}/R_{p}$ increases the setup’s ruggedness against laser fluctuations, vibrations and topography changes. In general it is possible to vary the illumination area with a simple optical setup adapting it to the desired operation conditions. Furthermore, since the optical setup only consists of a few inexpensive components, it offers great potential for industrial applications up to a 100% inline proof.

We were able to use the setup for other substrates with even further increased roughness due to electronic discharge texturing and thinner conversion coatings applied.

## Figures and Tables

**Table 1:** Overview of the process steps for the Cr–Zr coatings. The bold printed step 10 is different for each sample in a way presented in Table 2.

Processing Step	Function	Product	Concentration (Vol%)	Temperature (${}^{\circ }$C)	Time (min:sec)
1	Cleaning	SurTec^®^ 089	0.5	60	10:00
	Degreasing	SurTec^®^ 061	5.0		
2	Rinsing	Process water		RT	1:00
3	Rinsing	DI-water		RT	1:00
4	Alkaline Etching	SurTec^®^ 181	5.0	50	0:30
5	Rinsing	Process water		RT	1:00
6	Rinsing	DI-water		RT	1:00
7	Desmutting	SurTec^®^ 496	20	26	5:00
8	Rinsing	Process water		RT	1:00
9	Rinsing	DI-water		RT	1:00
**10**	**Passivation**	**SurTec^®^ 650 (SurTec^®^ 650 A)**	**20, (5)**	**Variable**	**Variable**
11	Rinsing	DI-water		RT	1:00
12	Drying	Oven		80	10:00

**Table 2:** Different passivation steps by altering the immersion time and temperature for each sample and the resulting coating weight *M*.

Sample	Temperature (${}^{\circ }$C)	Time (min:sec)	Coating Weight *M* $\left(\frac{g}{m^{2}}\right)$
**Ref**	-	-	-
**PT1**	30	1:00	0.114
**PT2**	40	1:00	0.204
**PT3**	30	3:00	0.363
**PT4**	40	3:00	0.413
**PT5**	30	5:00	0.473

**Table 3:** Roughness $R_{q}$ and peak-to-valley distance $D_{h}$ of the investigated cold-rolled aluminium samples.

Parameter	Ref	PT1	PT2	PT3	PT4	PT5
$R_{q}$ (μm)	0.29	0.26	0.26	0.29	0.26	0.28
$D_{h}$ (μm)	1.60	1.42	1.57	1.67	2.38	1.54

**Table 4:** Results of the X-ray photoelectron spectroscopy (XPS)-measurements performed on the six aluminium samples.

Sample	C (at%)	O (at%)	F (at%)	Al (at%)	Cu (at%)	Zr (at%)	Cr (at%)	N (at%)	S (at%)
**Ref**	24.1	45.4	2.2	27.9	0.3	-	-	-	-
**PT1**	43.0	37.0	5.0	2.9	-	6.3	3.4	2.0	0.6
**PT2**	42.3	38.0	4.6	1.8	-	6.8	3.5	2.3	0.7
**PT3**	39.7	36.9	7.0	2.4	-	7.1	4.0	2.4	0.7
**PT4**	41.2	37.5	6.1	2.3	-	7.0	3.8	1.4	0.6
**PT5**	40.6	37.3	6.9	3.1	-	7.2	3.4	1.1	0.5

**Table 5:** Overview of refractive indices and layer thicknesses of the TCC coatings determined by using a three layer model described in detail in Section 4.1.

Parameter	PT1	PT2	PT3	PT4	PT5
$n_{\mathrm{TCC}}^{\prime }$	≈1.94	2.06±0.25	1.52±0.25	1.23±0.01	1.21±0.01
$n_{\mathrm{TCC}}^{\prime \prime }$	0.005±0.005	0	0.0138±0.0050	0.0317±0.0049	0.0413±0.0023
*d* (nm)	36.0±5.9	47.8±2.3	68.5±0.5	71.1±0.3	70.4±0.4

**Figure A1:**
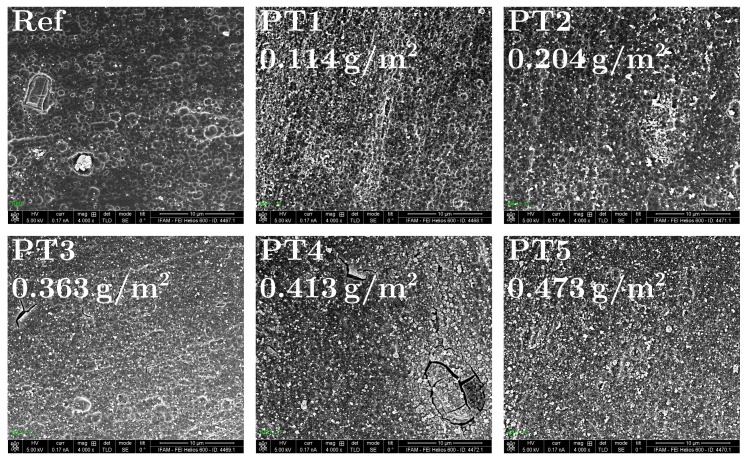
SEM images of all samples taken with a magnification of 4000 times are shown. An overview of the reference and coated specimens with applied coating weight is given.

**Figure A2:**
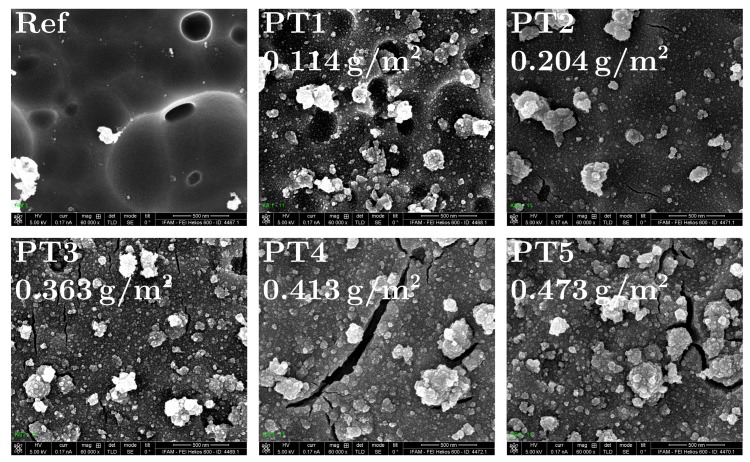
Investigation of the samples by means of SEM with a magnification of 60,000.

**Figure A3:**
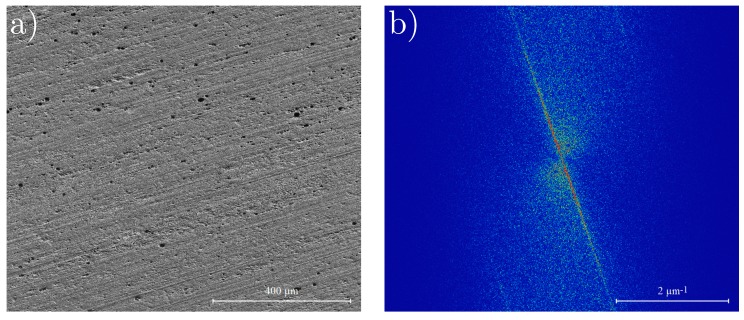
(**a**) An additional SEM image of the Ref sample and (**b**) the corresponding 2D-DFT.

**Figure A4:**
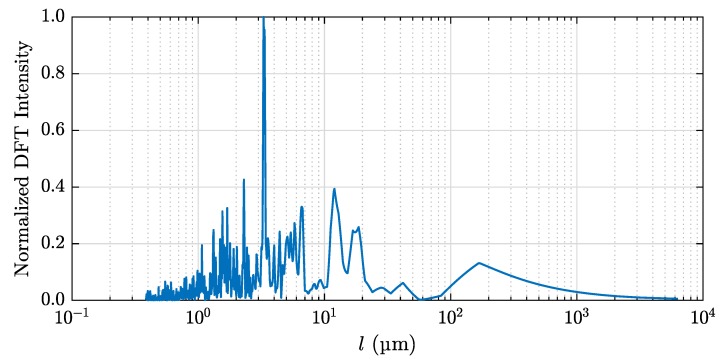
The frequencies along the pronounced line inside the 2D spectrum in Figure A3b are shown. The spectrum is normalized to 1 highlighting the most dominant frequency belonging to a groove distance of 3.3 μm.

## Supplementary Materials

The following are available online at https://www.mdpi.com/1424-8220/20/8/2164/s1.

## Abbreviations

The following abbreviations are used in this manuscript:

AA	aluminium alloy
BS	beam splitter
CCC	chromate conversion coating
D	Si-photodiode
FM	flip-mirror
L	lens
M	mirrors
MM	magnetic mirrors
P	polarizer
P-BS	polarizing beam splitter
PH	pinhole
PT	pretreatment
SE	spectroscopic ellipsometry
TCC	trivalent chromium conversion
TCP	trivalent chromaium coating

## Appendix A

The second part of the appendix deals with additional SEM-measurements leading to information about the in-plane structure, especially the distance between the grooves induced by the rolling process, already mentioned in Section 4.1. Figure A1 presents SEM images of all specimens taken with a magnification of 4000 times. The coated samples PT1-PT5 have a closed layer structure with observable cracks especially for increasing coating weight.

SEM images with an increased magnification of 60,000 times are shown in Figure A2. Just like before, cracks are clearly observable with a length up to ≈1μm and furthermore particulate deposits on the layer still covered by the TCC coating are noticeable.

In Figure A3a an additional SEM image of the Ref sample is shown. Pickling pits as well as grooves induced by the industrial rolling process are clearly distinguishable. Part b of Figure A3 depicts the corresponding 2D-DFT (discrete Fourier transform) of the SEM image with centered origin of the spectrum. Dominate frequencies are observable along a line that is rotated 20 ${}^{\circ }$ with respect to the vertical. These frequencies originate primary from the grooves orientated perpendicular to the line inside the spectrum.

For determining the distance between the grooves the normalized spectrum along the pronounced line is shown in Figure A4. The most dominant frequency belongs to a groove distance of 3.3 μm. Considering the peak-to-valley distance of $D_{h}=1.60\mu m$ (see Table 3) the neglecting of shadowing effects is reasonable for our samples.

## Appendix B

The final expression for the reflected, averaged, squared electric field $<|E_{r}{|}^{2}>$ is shown in Equation (A1) and composed of two summands, one for the diffusely scattered (underlined with “Diff”) and one for the specularly scattered light (underlined with “Spec”) [22].

(A1) <|Er|2>=E0AcosθinλR02·exp(-G)ρ02πT2F2A∑m=1∞Gmm!mexp-vxy2T24m︸Spec+πT2F2A∑m=1∞Gmm!mexp-vxy2T24m︸Diff

$E_{0}$ denotes the amplitude of the incoming electromagnetic plane wave striking the rough surface, A=2X·2Y the illuminated area in the shape of a rectangle, $\theta_{\mathrm{in}}$ the angle of incidence with respect to the surface normal, λ the wavelength of the incoming light, $R_{0}$ describes the distance between a detector and the surface and *T* the correlation length of the surface. The remaining factors are defined in the following:

(A2) $$\begin{array}{cc} \rho_{0} & =\mathrm{sinc}\left(v_{x}X\right)\;\mathrm{sinc}\left(v_{y}Y\right) \end{array}$$

(A3) $$\begin{array}{cc} G & ={\left(\frac{2\pi R_{q}}{\lambda }\left(\cos\theta_{\mathrm{in}}+\cos\theta_{\mathrm{out}}\right)\right)}^{2} \end{array}$$

(A4) $$\begin{array}{cc} F & =\left(\frac{1+\cos\theta_{\mathrm{in}}\cos\theta_{\mathrm{out}}-\sin\theta_{\mathrm{in}}\sin\theta_{\mathrm{out}}\cos\beta_{\mathrm{out}}}{\cos\theta_{\mathrm{in}}\left(\cos\theta_{\mathrm{in}}+\cos\theta_{\mathrm{out}}\right)}\right) \end{array}$$

(A5) $$\begin{array}{cc} v_{xy} & =\sqrt{v_{x}^{2}+v_{y}^{2}} \end{array}$$

(A6) $$\begin{array}{cc} v_{x} & =k(\sin\theta_{\mathrm{in}}-\sin\theta_{\mathrm{out}}\cos\beta_{\mathrm{out}}) \end{array}$$

(A7) $$\begin{array}{cc} v_{y} & =k(\sin\theta_{\mathrm{out}}\sin\beta_{\mathrm{out}}) \end{array}$$

Here, *k* is the magnitude of the wave vector resulting in k=2π/λ and $R_{q}$ denotes the rms roughness. Due to the detection used in our optical setup $\theta_{\mathrm{in}}=\theta_{\mathrm{out}}$ and $\beta_{\mathrm{out}}$ holds, leading to the following simplifications:

(A8) $$\begin{array}{cc} \rho_{0} & =1 \end{array}$$

(A9) $$\begin{array}{cc} G^{\prime } & ={\left(\frac{4\pi R_{q}}{\lambda }\;\cos\theta_{\mathrm{in}}\right)}^{2} \end{array}$$

(A10) $$\begin{array}{cc} F^{\prime } & =\left(\frac{1+\cos^{2}\theta_{\mathrm{in}}-\sin^{2}\theta_{\mathrm{in}}}{2\;\cos^{2}\theta_{\mathrm{in}}}\right)=1 \end{array}$$

(A11) $$\begin{array}{cc} v_{x} & =v_{y}=v_{xy}=0 \end{array}$$

By using the area $A_{0}$ irradiated under vertical incidence, the angle-dependent quantity *A* can be expressed as follows [23]:

(A12) $$\begin{array}{c} A=\frac{A_{0}}{\cos\theta_{\mathrm{in}}} \end{array}$$

Finally, by using the reflected power $I_{0}$ of a perfectly smooth conductive surface, the reflectance *R* can be derived:

(A13) R˜=IoutI0=exp(-G′)exp(-G′)πT2cosθinλ2∑m=1∞G′mm!m▵ω︸Spec+exp(-G′)πT2cosθinλ2∑m=1∞G′mm!m▵ω︸Diff

Here, Δω denotes the solid angle the measurement is performed. The polarization state of the incoming light is considered in the following way [23,24]:

(A14) $$\begin{array}{c} {\tilde{R}}_{s/p}=\tilde{R}\;\cdot \;R_{s/p} \end{array}$$

$R_{s}$ and $R_{p}$ denote the corresponding reflectance described by Fresnel for s- and p-polarization, respectively.

## References

[1] Becherini F., Lucchi E., Gandini A., Barrasa M.C., Troi A., Roberti F., Sachini M., Tuccio M.C.D., Arrieta L.G., Pockelé L. (2018). Characterization and thermal performance evaluation of infrared reflective coatings compatible with historic buildings. Build. Environ..

[2] Tschentscher J., Hochheim S., Brüning H., Brune K., Voit K.M., Imlau M. (2016). Optical Riblet Sensor: Beam Parameter Requirements for the Probing Laser Source. Sensors.

[3] Eggert J., Bourdon B., Nolte S., Rischmueller J., Imlau M. (2018). Chirp control of femtosecond-pulse scattering from drag-reducing surface-relief gratings. Photonics Res..

[4] Helmus M.N., Gibbons D.F., Cebon D. (2008). Biocompatibility: Meeting a Key Functional Requirement of Next-Generation Medical Devices. Toxicol. Pathol..

[5] Liu Z., Tabakman S., Welsher K., Dai H. (2009). Carbon nanotubes in biology and medicine: In vitro and in vivo detection, imaging and drug delivery. Nano Res..

[6] Kendig M., Davenport A., Isaacs H. (1993). The mechanism of corrosion inhibition by chromate conversion coatings from x-ray absorption near edge spectroscopy (Xanes). Corros. Sci..

[7] Zhao J. (1998). Corrosion Protection of Untreated AA-2024-T3 in Chloride Solution by a Chromate Conversion Coating Monitored with Raman Spectroscopy. J. Electrochem. Soc..

[8] Qi J., Hashimoto T., Walton J., Zhou X., Skeldon P., Thompson G.E. (2015). Formation of a Trivalent Chromium Conversion Coating on AA2024-T351 Alloy. J. Electrochem. Soc..

[9] Qi J.T., Hashimoto T., Walton J., Zhou X., Skeldon P., Thompson G. (2015). Trivalent chromium conversion coating formation on aluminium. Surf. Coat. Technol..

[10] Qi J., Walton J., Thompson G.E., Albu S.P., Carr J. (2016). Spectroscopic Studies of Chromium VI Formed in the Trivalent Chromium Conversion Coatings on Aluminum. J. Electrochem. Soc..

[11] Qi J., Gao L., Li Y., Wang Z., Thompson G.E., Skeldon P. (2017). An Optimized Trivalent Chromium Conversion Coating Process for AA2024-T351 Alloy. J. Electrochem. Soc..

[12] Qi J., Gao L., Liu Y., Liu B., Hashimoto T., Wang Z., Thompson G.E. (2017). Chromate Formed in a Trivalent Chromium Conversion Coating on Aluminum. J. Electrochem. Soc..

[13] Honselmann J., Volk P., Mankel E. (2015). Analyse der Schichtbildung Chrom(III)-haltiger Aluminium- Passivierungen. Galvanotechnik.

[14] Mujdrica Kim M., Kapun B., Tiringer U., Šekularac G., Milošev I. (2019). Protection of Aluminum Alloy 3003 in Sodium Chloride and Simulated Acid Rain Solutions by Commercial Conversion Coatings Containing Zr and Cr. Coatings.

[15] Dardona S., Jaworowski M. (2010). In situ spectroscopic ellipsometry studies of trivalent chromium coating on aluminum. Appl. Phys. Lett..

[16] Dardona S., Chen L., Kryzman M., Goberman D., Jaworowski M. (2011). Polarization Controlled Kinetics and Composition of Trivalent Chromium Coatings on Aluminum. Anal. Chem..

[17] Lehmann D., Seidel F., Zahn D.R. (2014). Thin films with high surface roughness: Thickness and dielectric function analysis using spectroscopic ellipsometry. SpringerPlus.

[18] Siah S., Hoex B., Aberle A. (2013). Accurate characterization of thin films on rough surfaces by spectroscopic ellipsometry. Thin Solid Film..

[19] Imlau M., Toschke Y., Rischmüller J., Schlag M., Brüning H., Brune K. (2019). Vorbehandlungsprüfung mit dem Laserpointer. JOT J. Für Oberflächentechnik.

[20] Davis J.R. (2001). Alloying: Understanding the Basics (06117G).

[21] Silvennoinen R., Peiponen K.E., Asakura T., Zhang Y.F., Gu C., Ikonen K., Morley E. (1992). Specular reflectance of cold-rolled aluminum surfaces. Opt. Lasers Eng..

[22] Beckmann P., Spizzichino A. (1987). The Scattering of Electromagnetic Waves from Rough Surfaces.

[23] Hensler D.H. (1972). Light Scattering from Fused Polycrystalline Aluminum Oxide Surfaces. Appl. Opt..

[24] Ragheb H., Hancock E.R. (2007). The modified Beckmann–Kirchhoff scattering theory for rough surface analysis. Pattern Recognit..

