# Supplementary material for: Inspection of Trivalent Chromium Conversion Coatings Using Laser Light: The Unexpected Role of Interference on Cold-Rolled Aluminium
Source: Sensors (Basel). 2020 Apr 11;20(8):2164. doi: 10.3390/s20082164 (PMC7218743; doi:10.3390/s20082164)
Supplement: Supplementary file 1 [file sensors-20-02164-s001.pdf]

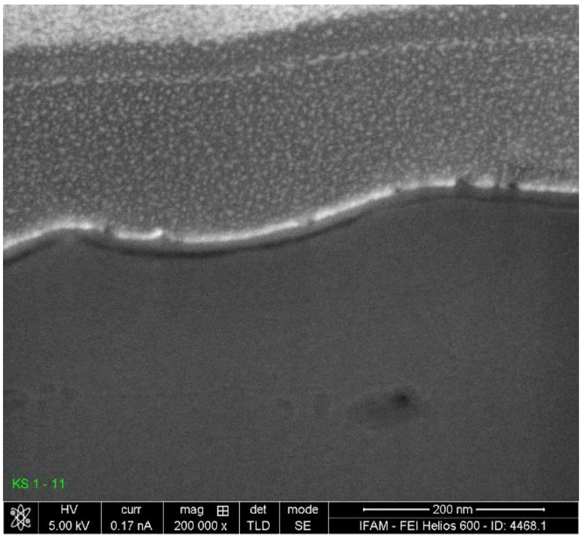

Figure 1: SEM, PT1

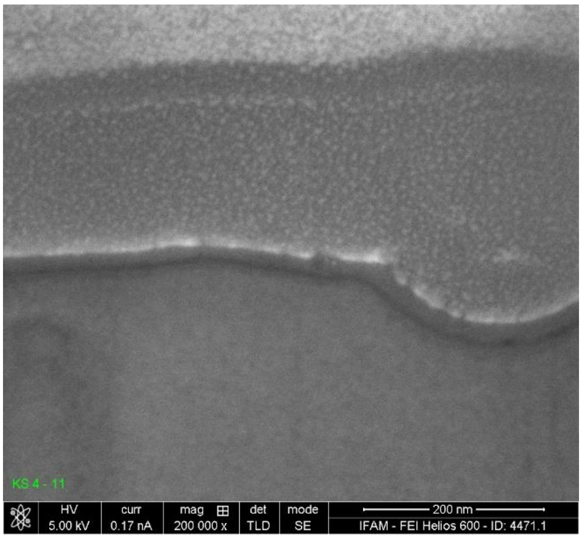

Figure 2: SEM, PT2

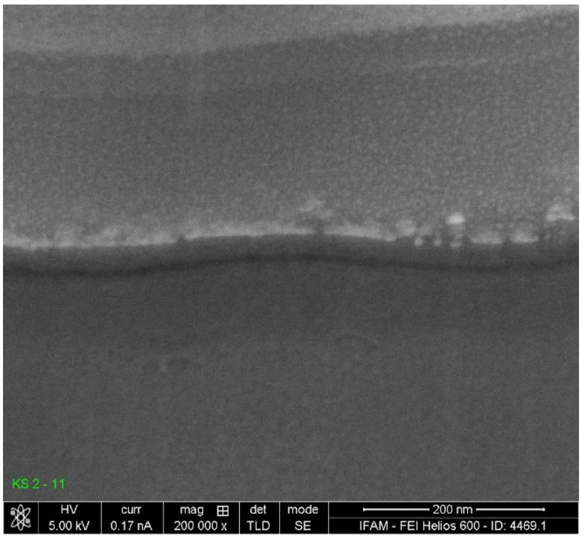

Figure 3: SEM, PT3

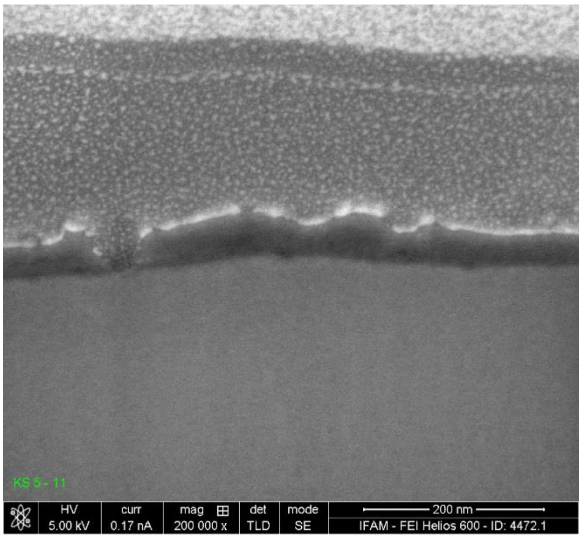

Figure 4: SEM, PT4

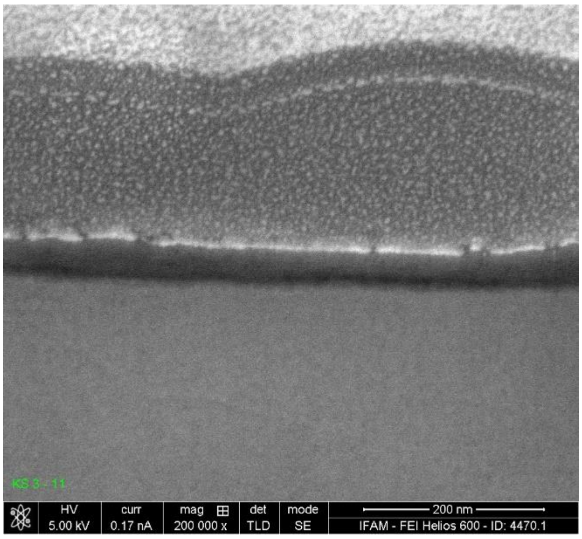

Figure 5: SEM, PT5

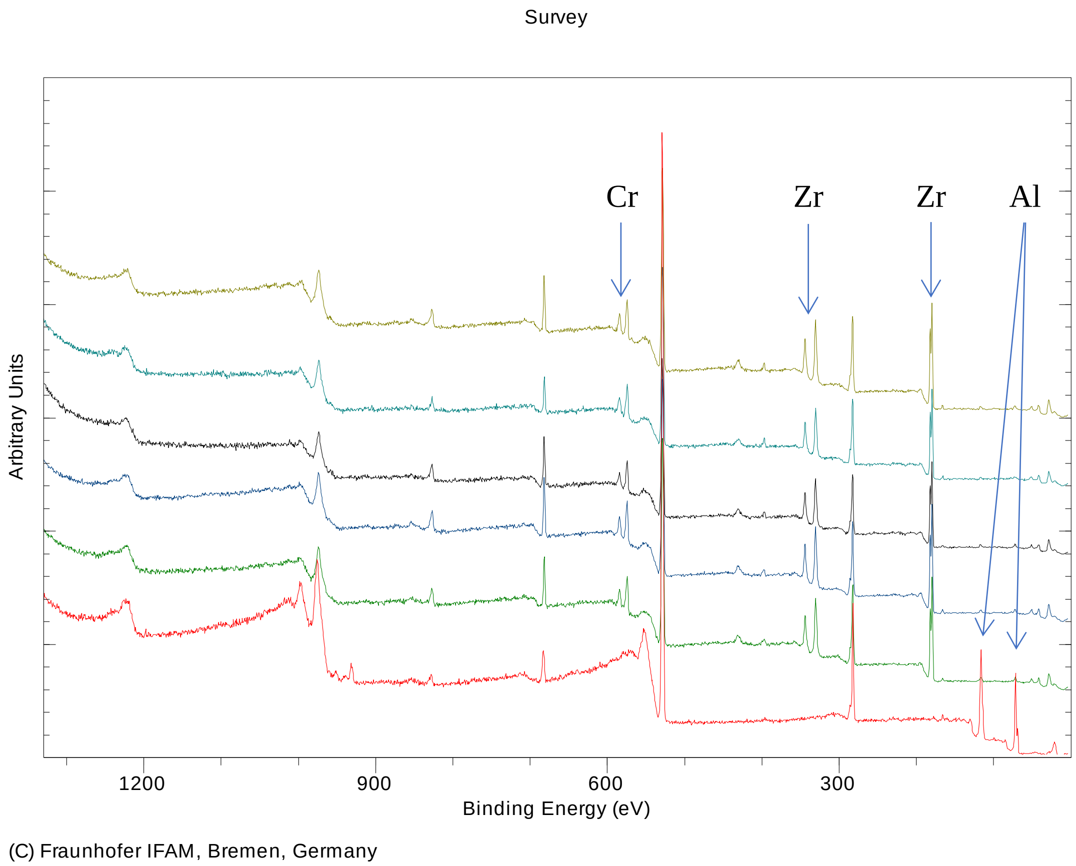

Figure 6: XPS, bottom-up ascending: Ref, PT1, PT3, PT5, PT2, PT4

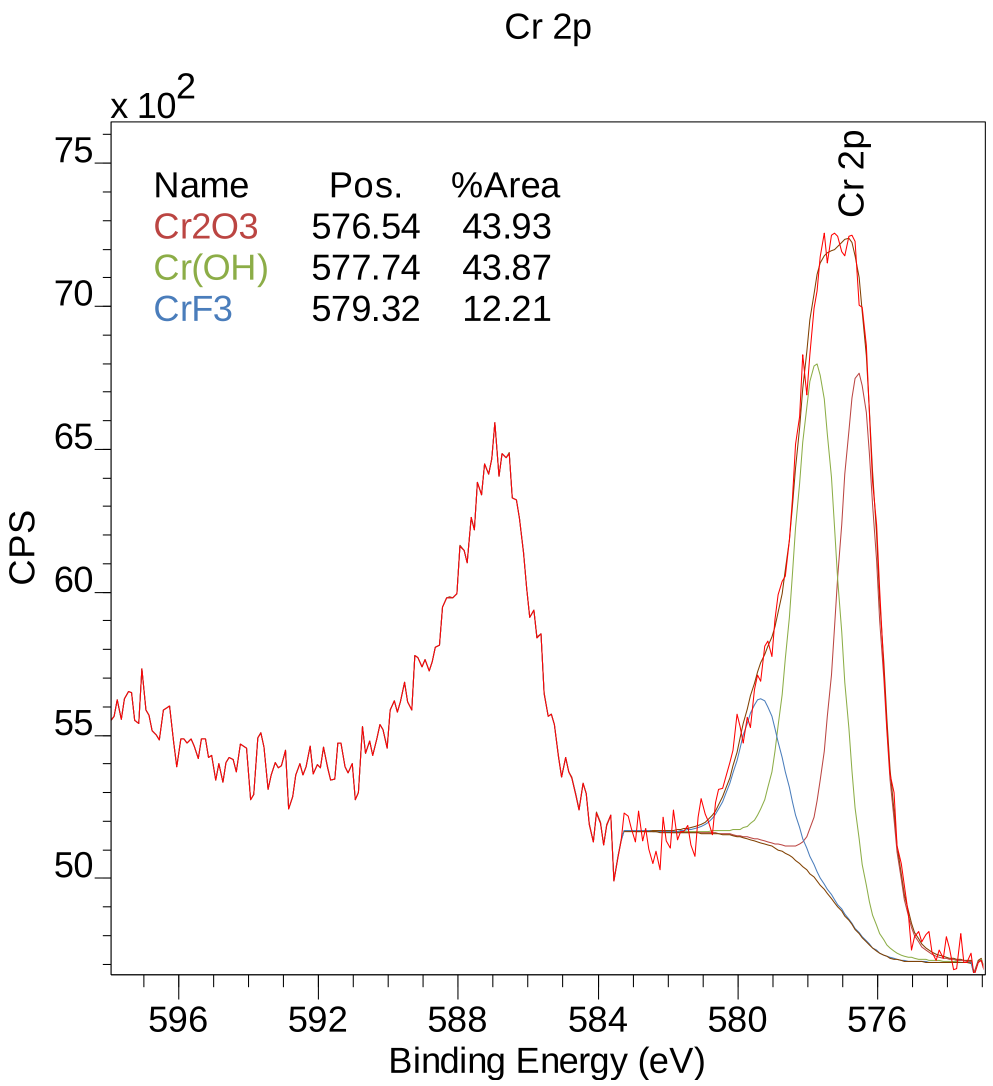

Copyright by Fraunhofer IFAM, Bremen

Figure 7: XPS, PT1, Pos. 1

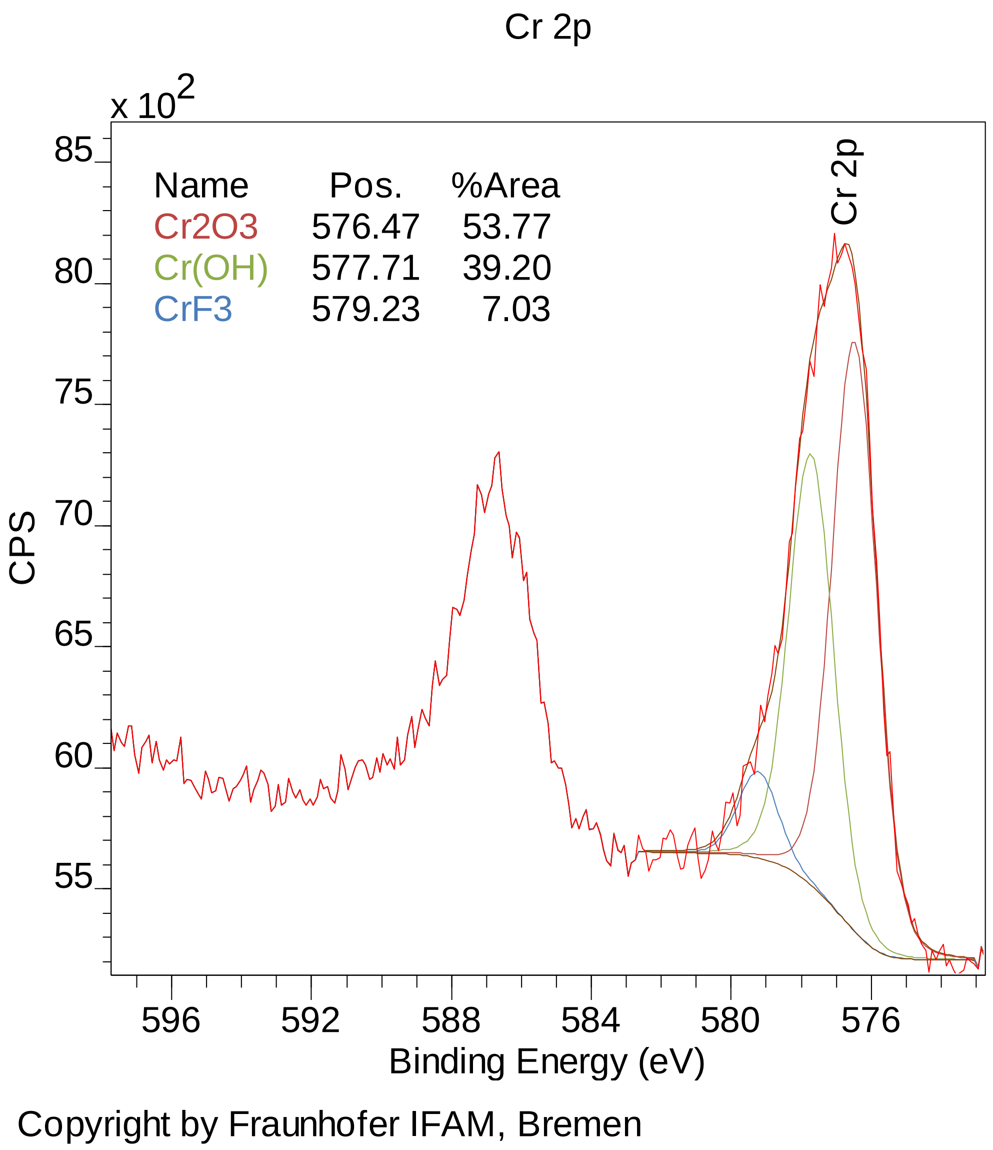

Figure 8: XPS, PT2, Pos. 1

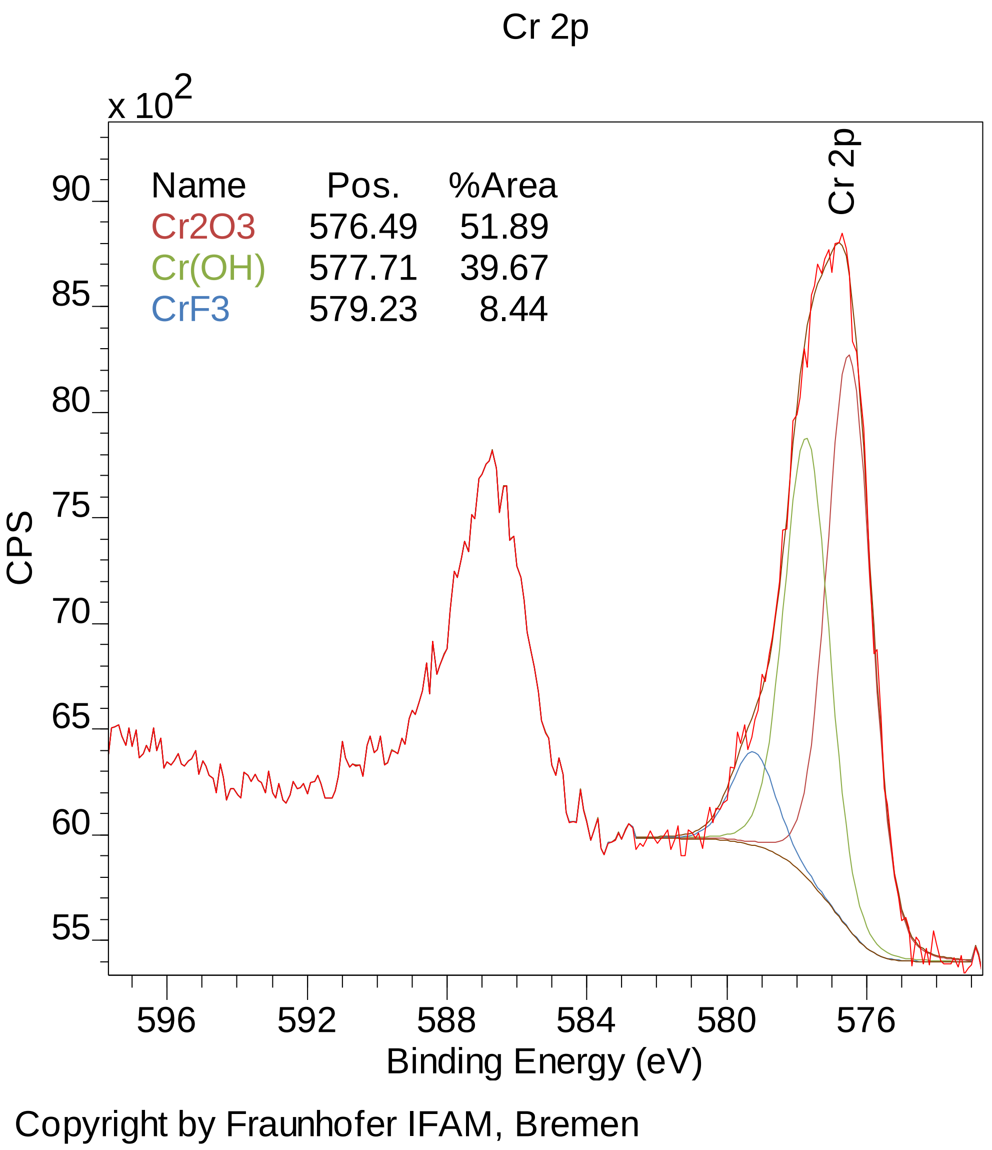

Figure 9: XPS, PT3, Pos. 2

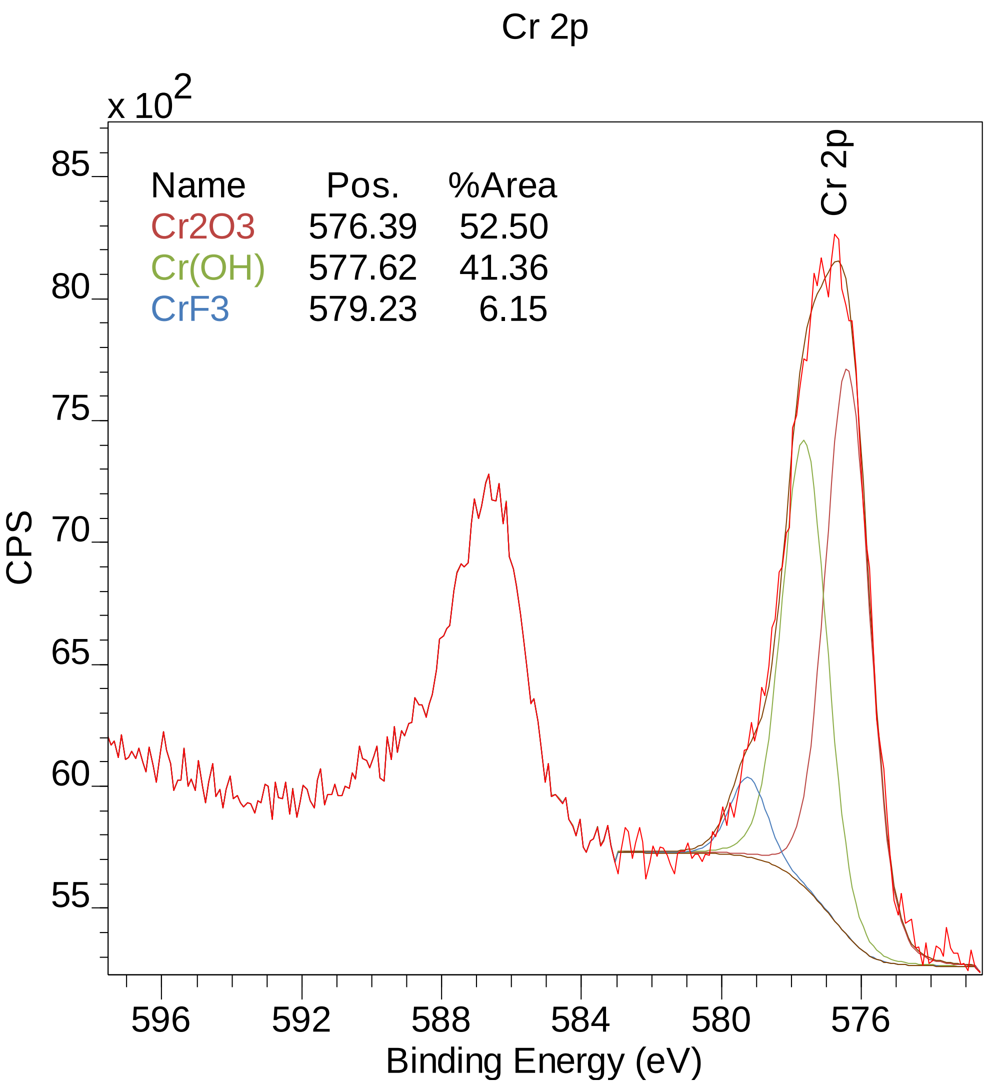

Copyright by Fraunhofer IFAM, Bremen

Figure 10: XPS, PT4, Pos. 1

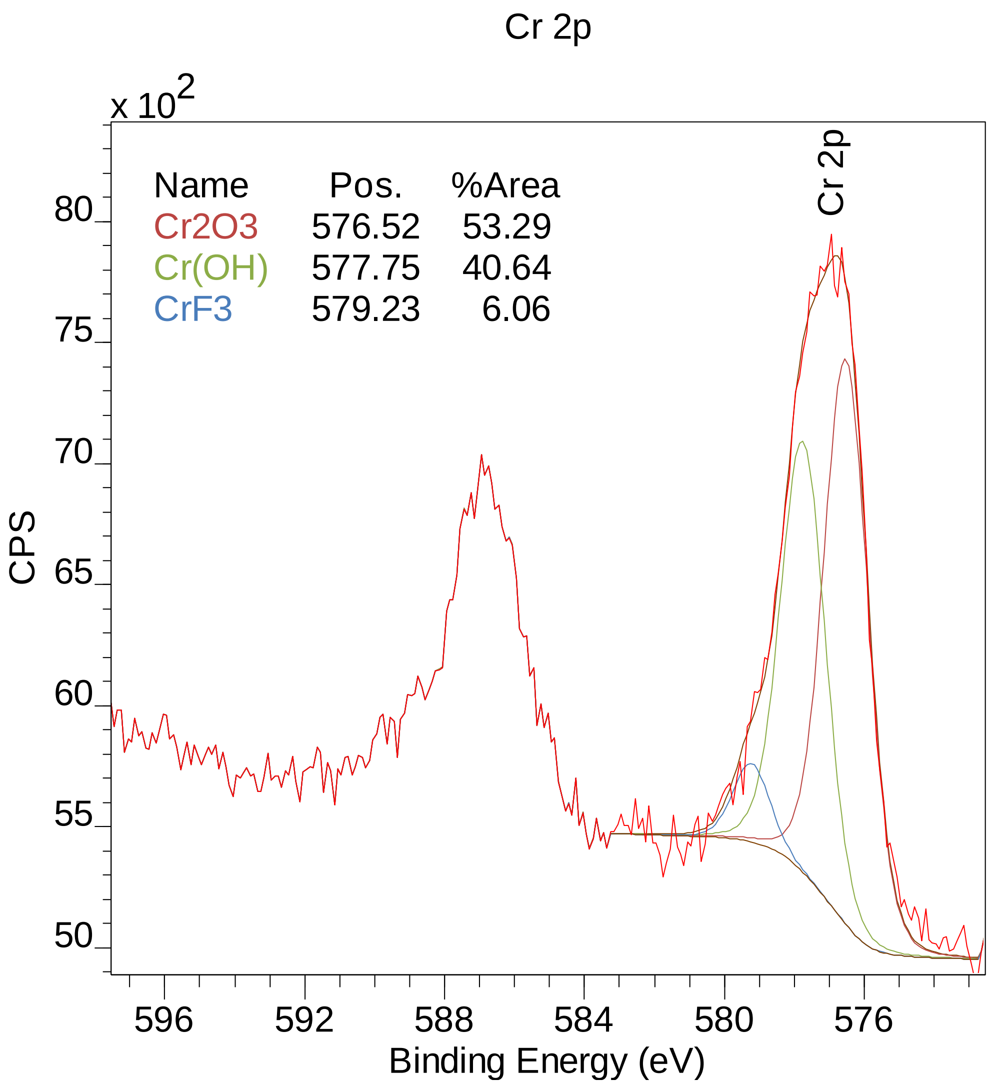

Copyright by Fraunhofer IFAM, Bremen

Figure 11: XPS, PT5, Pos. 1
